# Investigating the Impact of the TUITEK^®^ Patient Support Programme, Designed to Support Caregivers of Children Prescribed Recombinant Human Growth Hormone Treatment in Taiwan

**DOI:** 10.3389/fendo.2022.897956

**Published:** 2022-05-06

**Authors:** Pen-Hua Su, Sumaira Malik, Amrit Jheeta, Yen-Fan Lin, Su-Huei Su, Ekaterina Koledova, Selina Graham

**Affiliations:** ^1^ School of Medicine, Chung-Shan Medical University, Taichung City, Taiwan; ^2^ Department of Pediatrics and Genetics, Chung Shan Medical University Hospital, Taichung City, Taiwan; ^3^ Atlantis Healthcare, London, United States; ^4^ Medical Affairs Department, Merck Ltd., Taiwan, An Affiliate of Merck KGaA, Darmstadt, Germany, Taipei City, Taiwan; ^5^ Empowered Health, Global Medical Affairs Cardiometabolic and Endocrinology, Merck KGaA, Darmstadt, Germany; ^6^ Empowered Health, London, United Kingdom

**Keywords:** short stature, recombinant human growth hormone (r-hGH) treatment, adherence, caregiver, intervention

## Abstract

**Purpose:**

Poor adherence to recombinant human growth hormone (r-hGH) treatment presents a significant barrier to achieving optimal growth outcomes. It is important to identify and address the treatment adherence-related needs of children prescribed r-hGH treatment, and develop new approaches to improve adherence. We aimed to measure the impact of the TUITEK^®^ patient support programme, a multi-component personalized service intervention, on caregivers’ knowledge, beliefs, and perceptions of short stature and adherence to its treatment.

**Patients and Methods:**

The evaluation of the TUITEK^®^ patient support programme was conducted among 31 caregivers of children with short stature and receiving r-hGH treatment *via* the easypod™ auto-injector device in Taiwan. Caregivers within the ‘high risk’ category for knowledge, beliefs and perception factors influencing adherence to r-hGH treatment (disease and treatment coherence, emotional burden, self-administration, and treatment-related anxiety) were identified *via* the TUITEK^®^ personalization questionnaire and followed up with bi-weekly telephone calls by a nurse practitioner over a 3-month period. A Wilcoxon signed-rank test was used to compare changes in questionnaire-based scoring patterns between baseline and follow-up.

**Results:**

Between baseline and follow-up, the percentage of caregivers scoring as ‘high risk’ for emotional burden reduced by 37%; there was an improvement in confidence of self-administration by 57% and the percentage of caregivers scoring as ‘high risk’ for treatment-related anxiety reduced by 52%. At follow-up, all caregivers classified as ‘high risk’ within the disease and treatment coherence item at baseline moved into the ‘low risk’ category. Statistically significant changes in questionnaire scores between baseline and follow-up for disease and treatment understanding, emotional burden, self-administration, and treatment-related anxiety were also observed.

**Conclusion:**

These findings indicate that the TUITEK^®^ patient support programme can positively address disease and treatment-related barriers amongst caregivers regarding optimal adherence of their children to r-hGH treatment, which has the potential to positively impact on adherence levels and patient clinical health outcomes.

## Introduction

When sufficient levels of growth hormone (GH) are not produced or secreted naturally in early childhood, daily supplementary bio-synthetic injections as a prescriptive treatment have been used for many years to replace and replenish the deficient hormone (1, 2). Recombinant human growth hormone (r-hGH) known also as somatropin is a recombinant form of human GH, manufactured by recombinant DNA technology (3, 4). The structure of the bio-synthetic growth hormone used in treatment is identical to the growth hormone produced naturally by the pituitary gland (3, 4). The prescription of r-hGH has now been approved and licensed to treat several growth-related indications, which include Growth Hormone Deficiency (GHD), Turner Syndrome (TS) Small for Gestational Age (SGA) with failure to catch up, Idiopathic Short Stature (ISS), Russell Silver Syndrome (RSS), Prader-Willi Syndrome (PWS) Short stature HomeobOX-containing gene (SHOX) deficiency, Chronic Renal Insufficiency (CRI) and Noonan Syndrome (NS) (2). While, r-hGH treatment offers a well-established treatment for children and adolescents with a short stature and has been found to significantly improve height outcomes potential (1, 5–8), poor adherence to treatment presents a significant barrier to achieving optimal outcomes and carries an appreciable economic burden (9–13). It was reported within a systematic review that up to 82% of children and adolescents treated with r-hGH treatment and their families were non-adherent to treatment as prescribed (14).

Research suggests that managing r-hGH treatment can be hugely demanding for the patient as well as their families. Several studies have identified a range of determinants of non-adherence to r-hGH treatment (14). Potentially modifiable factors associated with non-adherence have been reported to include knowledge and understanding of the condition, discomfort and pain associated with daily injections, lack of understanding of the consequences of missed r-hGH doses, poor administration technique, forgetting to administer the medication and inadequate contact with the health care professional (HCP) and the quality of the HCP-patient relationship (14–16).

Given the effects of non-adherence on patients and their families, HCPs, and the health care system, it is evident that treatment non-adherence is an important health issue (13). It is imperative that both research and clinical practice identify the adherence-related needs of children prescribed with r-hGH treatment and their families and develop new ways in which to improve adherence.

In view of this, patient support programmes (PSP) have been designed to support patients and families to better manage the disease and complex treatment regimens, gain the full clinical benefits of the treatment, improve medication adherence, and reduce complications and related costs. These programmes have demonstrated improved outcomes in a wide variety of diseases through such strategies as individual and group medication support and multidisciplinary health care team training and coaching (17–22).

The TUITEK^®^ PSP is a multicomponent digital, personalized programme, designed to support the needs of patients, caregivers, and HCPs throughout the different phases of the child’s treatment care pathway. The intervention comprises of two key service components: 1) a PSP training session, which aims to provide the PSP nurses with the tools and strategies required to deliver the TUITEK^®^ patient support programme, and 2) a PSP Manual, for the PSP nurses’ use, which consists of A) a personalization screener, to identify key issues and challenges faced by patients and caregivers and tailor the content of programme; and B) a set of personalized one-to-one telephone call guide and resource packs which utilize a range of behavior change technique (BCTs), to be used by PSP nurses during scheduled outbound calls with caregivers. Nurse-led calls which use behavior change techniques and implement motivational interviewing principles have been shown to affect meaningful behavior change, across different health conditions such as smoking cessation, increasing physical activity and improving diet (23–25) as well as demonstrating a positive impact on treatment adherence (26, 27).

The key objective was to measure the impact of the TUITEK^®^ PSP on the knowledge, beliefs, and perceptions of caregivers with children receiving r-hGH (somatropin, Merck KGaA, Darmstadt, Germany).

## Methods

A prospective, observational design was conducted to measure the impact of the PSP for caregivers of children where r-hGH had been indicated. Ethical approval was granted by Chung Shan Medical University Hospital Institutional Review Board.

### Participant Recruitment and Selection

A 3-month evaluation of the TUITEK^®^ patient support programme was conducted among 31 caregivers of children receiving r-hGH treatment *via* the easypod™ auto-injector device in Taiwan.

Participants were randomly recruited from Chung Shan Hospital, Taiwan between January, and April 2020. Caregivers were eligible if they were the caregiver (mother/father/immediate caregiver) of a child indicated for r-hGH treatment and between the ages 25-60 years. Participants suffering from any mental illness were excluded. Eligible participants were invited to participate. Written informed consent was obtained from each participant before the commencement of the programme. All caregivers approached during the recruitment period were recruited, continued to the end of the 3-month evaluation period and completed the follow-up assessment.

### Data Collection

Eligible caregivers were contacted by a nurse practitioner *via* telephone to complete an initial bespoke set of personalization questions exploring knowledge, beliefs and perceptions surrounding their child’s treatment (see **Supplementary Table 1**). Based on the participants’ response of the baseline personalization questionnaire, a personalization algorithm was used to surface ‘high risk’ topics for discussion in the subsequent interventional calls. Each factor-based interventional call (see **Supplementary Tables 2, 3**), administered by the nurse practitioner, focused on a single priority topic. Factor-based calls covered four key factor topics: Disease and Treatment Coherence; Emotional burden; Treatment-Related Anxiety; and Self-Administration. If no scores fell into the high risk cut off range, no interventional calls were scheduled. The number and order of each interventional call were tailored to the needs of each caregiver based on the responses to the personalization questionnaire and immediate areas that required attention.

The interventional telephone calls with the nurse practitioner were scheduled to occur bi-weekly with caregivers over a 3-month period. With use of the structured interventional call guides (see **Supplementary Table 2**) and motivational interviewing principles, the nurse practitioner was able to support the caregiver in addressing the high-risk factors identified and facilitate positive change in knowledge, beliefs, perceptions, and behavior, tailored to the caregivers’ needs (see **Supplementary Table 3**). Two weeks after the final interventional call, participants were contacted by the nurse practitioner *via* telephone to complete a follow-up personalization questionnaire (see **Supplementary Table 1**), to determine any changes in knowledge, beliefs, and perceptions regarding their child’s treatment.

### Statistical Analysis

Data entry, management and statistical analyses were conducted using version 25.0 (IBM Corp., Armonk, N.Y., USA). All participant data were included within the analyses.

Descriptive statistics were used initially to summarize caregiver demographics, caregiver scores on the questionnaire items and the proportion of people scoring high risk at each time point. Data from the baseline and post follow-up personalization questionnaire were used to examine the scoring pattern of caregiver knowledge, beliefs, and perceptions. As the distribution of the sample was non-parametric (non-normal distribution), a Wilcoxon signed-rank test was used to compare changes in questionnaire-based scores between paired groups at baseline and follow-up. A *p* value of ≤.05 was considered statistically significant.

## Results

### Demographic Characteristics

A total of 31 caregivers participated in the programme. Reasons for child initiation of r-hGH treatment included ISS (81%); SGA (10%); GHD (6%) and TS (3%) and all were receiving r-hGH at the start of the programme.

### Caregivers’ Knowledge, Beliefs, and Perception Factors

The results revealed that between baseline and follow-up, the percentage of caregivers scoring as high risk for emotional burden reduced by 37%; there was also a positive change in confidence of self-administration by 57% and the percentage of caregivers scoring as high-risk for treatment-related anxiety was reduced by 52%. At follow-up, all caregivers classified as ‘high risk’ within the disease and treatment coherence category at baseline had moved into the ‘low risk’ category. **Table 1** presents an overview of these findings. Furthermore, statistically significant positive changes in average questionnaire scores for disease and treatment coherence, emotional burden, self-administration, and treatment-related anxiety were observed between baseline and follow-up (see **Table 1**).

**Table 1:** Changes in scores from baseline to follow-up.

Factor	Baseline		Follow-up		Mean Difference	Percentage Change (%)
	n (%)	Mean	n (%)	Mean		
**Disease and Treatment Coherence**	11 (35)	4.1	0 (0)	4.7	0.6*	100.0
**Emotional Burden**	27 (87)	3.7	17 (55)	2.6	-1.0*	37.0
**Treatment-related anxiety**	25 (81)	3.5	12 (39)	2.4	-1.1*	52.0
**Self-administration**	28 (90)	1.9	12 (39)	3.4	1.5*	57.0

*p <.01.

## Discussion

Adherence to prescribed r-hGH treatment is vital to achieve the maximal therapeutic benefit and improve clinical health outcomes (1, 5–8). It is therefore important to support HCPs to identify and manage the adherence-related issues of children prescribed with r-hGH treatment and their families. The TUITEK^®^ patient support programme was designed to support the needs of patients, caregivers, and healthcare professionals throughout the different phases of the child’s treatment care pathway. The impact of the brief, tailored, one-to-one telephone-support intervention was measured, specifically on caregivers’ disease and treatment knowledge, beliefs, and perceptions.

Overall, our evaluation demonstrated a positive shift in caregiver knowledge, beliefs, and perceptions; caregivers showed improved understanding of disease and treatment, a reduced experience of emotional burden, improved confidence relating to the transition of parental to child administration and reduced treatment-related anxiety.

The factors identified [disease and treatment coherence, emotional burden, self-administration, and treatment-related anxiety] correspond with the wider literature, which has identified factors associated with non-adherence to r-hGH treatment (5, 9, 14, 28–30) and in addition explored the views, perceptions, and experiences of parents/caregivers of children receiving r-hGH treatment (31–33).

The lack of understanding of the condition (particularly the chronicity of the disease) and treatment amongst r-hGH literature have emerged as important reasons for treatment non-adherence (1, 14–16, 29). Thus, ensuring that both patients and caregivers have a good coherent understanding of the disease, and its treatment is therefore crucial in achieving optimal growth outcomes. Although, disease and treatment understanding in this sample of caregivers was relatively high at baseline, results show that perceived understanding of the disease and of treatment improved for all patients after the intervention. This therefore suggests that the brief, tailored nurse support intervention may be used as an appropriate avenue of support in which to address this important factor.

Moreover, many caregivers reported high levels of treatment anxiety at baseline, suggesting that this is a significant challenge for people caring for a child with short stature. Indeed, broader literature on the impact of short stature has reported that treatment anxiety is a common issue particularly for caregivers (15, 30, 33–35). For example, a large-scale qualitative exploration of narratives found that parents experienced persistence worries about potential unknown side effects of therapy and had a need for more reassurance about their child’s future (34). Furthermore, in a survey of parents caring for a child on r-hGH treatment, 83% reported that they would appreciate psychological support to overcome their anxiety around treatment administration (33). As trained psychological support might be not readily available elsewhere, PSP programs with enhanced personalization features based on motivational interviewing principles could be appropriate and efficient alternative to be considered. Our evaluation revealed that treatment anxiety significantly improved at follow-up, indicating that this interventional support can be an effective method towards addressing these support needs.

Reducing caregiver anxiety around treatment administration may in turn help to increase their comfort level in allowing their child to self-administer the injection. Research from various pediatric conditions has shown that parents/caregivers often feel anxious or reluctant to hand over treatment control to their child (36). As has been reported previously (31–33), empowering patients to involve themselves in their own healthcare from a young age is important and can help to tackle non-adherence, as well as establish effective self-management behaviors for when patients reach puberty and begin to transition to adult care. Results from our evaluation showed that caregiver confidence in the patient self-administering their treatment increased significantly at follow-up, thus indicating that the intervention can positively impact both treatment-anxiety for the caregiver and perceptions around patient self-administration.

Lastly, a key area assessed through our evaluation related to the emotional burden of the disease. Short stature can have a significant impact on the psychosocial functioning and quality of life for both patients and caregivers. Research has shown that parents of children with short stature report persistent worries about their child’s physical and emotional well-being (16, 31, 32, 34). More broadly, studies across chronic pediatric endocrine conditions have revealed that distress associated with feelings of guilt, worry and fear are common amongst caregivers (35, 36), which can impact upon adherence behaviors. A strong emotional burden of caring for a child treated with r-hGH treatment was also evident in this sample as demonstrated by high baseline scores on this factor; this significantly reduced as a result of the intervention. This indicates that offering interventional emotional support and guidance to parents/caregivers during this period is thus important.

Overall, our findings reveal that the brief, personalized, one-to-one telephone-support intervention, delivered by PSP nurse practitioners can positively address and manage some of the disease and treatment-related barriers to optimal adherence amongst caregivers of children prescribed with r-hGH treatment, further establishing the TUITEK^®^ PSP as a feasible and replicable intervention with favorable outcomes.

### Limitations and Directions for Future Research

The findings should be interpreted within the context of its limitations. Firstly, the evaluation used a small select sample of caregivers from a single center in Taiwan, which challenged the generalizability of the findings. Secondly, the nurse support calls delivered were bi-weekly and limited to a short follow-up of 3 months. It is therefore unclear if the observed changes in beliefs demonstrated were sustained over the long-term. To further contribute towards the work supporting the use of r-hGH treatment, it is recommended that future studies, where possible, include longitudinal analyses with larger samples, which would increase the strength and generalizability of the findings. Lastly, to monitor adherence was beyond the scope of this evaluation, therefore it was not possible to ascertain whether the TUITEK^®^ PSP would lead to improving r-hGH adherence. Due to the importance of this health-related behavior in improving long-term health outcomes, it is recommended that this is addressed within future research.

## Conclusion

Given the known implications of treatment non-adherence on the patient, HCPs, and the health care system, it is evident that non-adherence to r-hGH treatment is an increasingly important health issue that requires necessary attention. The TUITEK^®^ PSP provides HCPs with a simple, practical, and useful method in which to improve condition and treatment-related knowledge, perceptions, and beliefs among caregivers of children with growth hormone deficiencies and better support their adherence-related needs. We offer that this intervention requires further testing and evaluation by way of a randomized controlled trial in order to determine the efficacy of the TUITEK^®^ programme on patient, caregiver, and HCP beliefs, as well as behavior and clinical health outcomes over the long-term and across multiple regions.

## Data Availability Statement

The datasets presented in this article are not readily available because this is a prospective, observational design study. Requests to access the datasets should be directed to EK: ekaterina.koledova@merckgroup.com.

## Ethics Statement

Ethical approval was granted by Chung Shan Medical University Hospital Institutional Review Board. Written informed consent was obtained from each participant before the commencement of the evaluation.

## Author Contributions

P-HS, S-HS, and Y-FL contributed to the work reported, in the conception, design and execution of the evaluation. SM and AJ contributed through drafting the article, in addition to acquisition of data, analysis and interpretation. SG contributed to the work, through writing, drafting and substantially revising the article. EK revised the articles critically for important intellectual content, agreed on all versions of the article and agreed on the journal to which the article has been submitted. All authors agree to be accountable for all aspects of the work.

## Funding

This work has been funded by Merck (CrossRef Funder ID: 10.13039/100009945). The funding organization has played a role in the design, the preparation of the manuscript and in the decision to submit the manuscript for publication.

## Conflict of Interest

P-HS and S-HS are employees of Chung Shan Medical University Hospital and received funding from Merck KGaA, Darmstadt, Germany to carry out this work. AJ and SM are employees of Atlantis Healthcare, London, UK and received funding from Merck KGaA, Darmstadt, Germany to carry out this work. Y-FL is an employee of Merck Ltd., Taiwan, an affiliate of Merck KGaA, Darmstadt, Germany. SG is a consultant to Atlantis Healthcare. EK is an employee of Merck KGaA, Darmstadt, Germany and holds shares in the company.

## Publisher’s Note

All claims expressed in this article are solely those of the authors and do not necessarily represent those of their affiliated organizations, or those of the publisher, the editors and the reviewers. Any product that may be evaluated in this article, or claim that may be made by its manufacturer, is not guaranteed or endorsed by the publisher.
