# Supplementary material for: Investigating the Impact of the TUITEK® Patient Support Programme, Designed to Support Caregivers of Children Prescribed Recombinant Human Growth Hormone Treatment in Taiwan
Source: Front Endocrinol (Lausanne). 2022 May 6;13:897956. doi: 10.3389/fendo.2022.897956 (PMC9120661; doi:10.3389/fendo.2022.897956)
Supplement: Supplementary file 1 [file Table_1.docx]

**Supplementary Table 1 Personalization questions and scoring**

| **Factor** | | **Question** | **Scoring** | **Cut off ‘High Risk’** |
| --- | --- | --- | --- | --- |
| **1** | **Disease and Treatment Coherence** | How well do you feel you understand your child’s treatment and condition? | **1.** Don’t understand at all  **2.** Understand a little  **3.** Understand somewhat  **4.** Mostly understand  **5.** Understand very clearly | If the score is **1-3,** the caregiver receives a Disease and Treatment Coherence call. |
| **2** | **Emotional burden** | How much does your child’s condition affect you emotionally? (eg, does it make you angry, guilty or frustrated?) | **1.** Not at all  **2.** Slightly  **3.** Moderately  **4.** Very  **5.** Extremely | If the score is **3-5,** the caregiver receives an Emotional burden call. |
| **3** | **Treatment-related anxiety** | How much does your child’s treatment worry you? (eg, do you feel worried about side effects or about giving injections, if applicable) | **1.** Not at all  **2.** Slightly  **3.** Moderately  **4.** Very  **5.** Extremely | If the score is **3-5,** the caregiver receives a Treatment-related anxiety call. |
| **4** | **Self-administration** | How comfortable do you feel giving your child responsibility over managing their condition and treatment? | **1.** Not at all comfortable  **2.** Slightly comfortable  **3.** Moderately comfortable  **4.** Very comfortable  **5.** Extremely comfortable | If the score is **1-3,** the caregiver receives a Self-administration call. |
