# Supplementary material for: Investigating the Impact of the TUITEK® Patient Support Programme, Designed to Support Caregivers of Children Prescribed Recombinant Human Growth Hormone Treatment in Taiwan
Source: Front Endocrinol (Lausanne). 2022 May 6;13:897956. doi: 10.3389/fendo.2022.897956 (PMC9120661; doi:10.3389/fendo.2022.897956)
Supplement: Supplementary file 2 [file Table_2.docx]

**Supplementary Table 2** Interventional Telephone Call Structure

| **Step** | **Description** |
| --- | --- |
| **Greeting** | Nurse will introduce/reintroduce themselves. |
| **Follow-up** | Nurse will follow up on previous call topic (if applicable). |
| **Explain purpose**  **of call** | Nurse will explain the purpose/objective of the call. |
| **Explore and Change** | Nurse will probe to understand how the caregiver is impacted and how they are coping currently and signpost the caregiver to relevant resources based on their responses. |
| **Set homework activity** | Nurse will set homework activity to encourage caregiver to put relevant strategies into practice before the next call. |
| **Closing:** | Nurse will summarize key points of the point, will ask caregiver if they have any questions or concerns and will confirm date and time for next appointment. |
