# Supplementary material for: Investigating the Impact of the TUITEK® Patient Support Programme, Designed to Support Caregivers of Children Prescribed Recombinant Human Growth Hormone Treatment in Taiwan
Source: Front Endocrinol (Lausanne). 2022 May 6;13:897956. doi: 10.3389/fendo.2022.897956 (PMC9120661; doi:10.3389/fendo.2022.897956)
Supplement: Supplementary file 3 [file Table_3.docx]

**Supplementary Table 3: Call guide outline**

| **Factor-based Call Guide** | **Call objectives** | **Recommended Interventional Resources** | **Recommended Behavior Change Techniques (BCTs):** |
| --- | --- | --- | --- |
| **Disease and Treatment Coherence** | - To build and improve understanding of their condition. - To build and improve understanding of treatment. - To help caregiver to make sense of the disease in context of different experiences. - To manage expectations. - To promote adherence and monitoring of adherence. | - GHD – The facts - Saizen – The facts - Managing Thinking Traps | - Credible source - Information about health consequences - Monitoring of behavior by others with/without feedback - Framing/re-framing - Reducing negative emotions |
| **Emotional burden** | - To build confidence in supporting the child with any emotional challenges. - To support to manage emotions such as frustration, worry, stress. - To support to build social support. | - Seeking support - Managing Thinking Traps - Managing Stress - Managing Mood | - Social support - Framing/re-framing - Reducing negative emotions - Information about health consequences/ Information about emotional consequences - Instruction on how to perform a behavior - Behavioural practice/rehearsal - Self-monitoring of outcomes of a behavior |
| **Treatment-related anxiety** | - To ensure sound understanding of Saizen. - To build confidence giving Saizen. - To address any treatment-related concerns. | - Saizen® – The Facts - Boosting Confidence - Managing Thinking Traps - Making Life Easier Through Problem Solving | - Credible source - Information about health consequences - Monitoring of behavior by others with/without feedback - Framing/re-framing - Reducing negative emotions - Focus on past success - Self-talk - Problem Solving - Action Planning |
| **Self-administration** | - Support caregiver to develop their child’s responsibility and problem solve any challenges with administration. - Support to manage emotions around ‘letting go’ of treatment. | - Seeking Support - Boosting Confidence - Making Life Easier Through Problem Solving | - Social support - Focus on past success - Self-talk - Problem Solving - Action Planning |
